# Variation in Phenotype, Parasite Load and Male Competitive Ability across a Cryptic Hybrid Zone

**DOI:** 10.1371/journal.pone.0005677

**Published:** 2009-05-25

**Authors:** Devi Stuart-Fox, Raquel Godinho, Joëlle Goüy de Bellocq, Nancy R. Irwin, José Carlos Brito, Adnan Moussalli, Pavel Široký, Andrew F. Hugall, Stuart J. E. Baird

**Affiliations:** 1 Department of Zoology, University of Melbourne, Melbourne, Australia; 2 CIBIO – Centro de Investigação em Biodiversidade e Recursos Genéticos da Universidade do Porto, Vairão, Portugal; 3 Department of Biology, University of Antwerp, Antwerp, Belgium; 4 Department of Biology, Univeristy of York, York, United Kingdom; 5 Sciences Department, Museum Victoria, Victoria, Australia; 6 Department of Biology and Wildlife Diseases, Faculty of Veterinary Hygiene and Ecology, University of Veterinary and Pharmaceutical Sciences Brno, Brno, Czech Republic; 7 CEBB, School of Earth and Environmental Sciences, University of Adelaide, Adelaide, Australia; 8 INRA, CBGP, Campus international de Baillarguet, Montferrier-sur-Lez, France; University of Bristol, United Kingdom

## Abstract

**Background:**

Molecular genetic studies are revealing an increasing number of cryptic lineages or species, which are highly genetically divergent but apparently cannot be distinguished morphologically. This observation gives rise to three important questions: 1) have these cryptic lineages diverged in phenotypic traits that may not be obvious to humans; 2) when cryptic lineages come into secondary contact, what are the evolutionary consequences: stable co-existence, replacement, admixture or differentiation and 3) what processes influence the evolutionary dynamics of these secondary contact zones?

**Methodology/Principal Findings:**

To address these questions, we first tested whether males of the Iberian lizard *Lacerta schreiberi* from two highly genetically divergent, yet morphologically cryptic lineages on either side of an east-west secondary contact could be differentiated based on detailed analysis of morphology, coloration and parasite load. Next, we tested whether these differences could be driven by pre-copulatory intra-sexual selection (male-male competition). Compared to eastern males, western males had fewer parasites, were in better body condition and were more intensely coloured. Although subtle environmental variation across the hybrid zone could explain the differences in parasite load and body condition, these were uncorrelated with colour expression, suggesting that the differences in coloration reflect heritable divergence. The lineages did not differ in their aggressive behaviour or competitive ability. However, body size, which predicted male aggressiveness, was positively correlated with the colour traits that differed between genetic backgrounds.

**Conclusions/Significance:**

Our study confirms that these cryptic lineages differ in several aspects that are likely to influence fitness. Although there were no clear differences in male competitive ability, our results suggest a potential indirect role for intra-sexual selection. Specifically, if lizards use the colour traits that differ between genetic backgrounds to assess the size of potential rivals or mates, the resulting fitness differential favouring western males could result in net male-mediated gene flow from west to east across the current hybrid zone.

## Introduction

The study of hybrid zones has led to important advances in understanding the nature of reproductive isolation [1]–[3]. Most well-characterized hybrid zones involve lineages with obvious phenotypic differences and some degree of pre- or post-zygotic isolation [2], [4]. However, phylogeographic studies are revealing an increasing number of zones of secondary contact between lineages that are deeply genetically divergent yet morphologically cryptic [5], that is, only distinguished with the hindsight of genetic information. In most cases, whether the lineages have diverged in subtle aspects of their morphology, physiology or behaviour and whether there exist any barriers to gene flow is not known; yet this information is important for predicting the evolutionary consequences of secondary contact: stable co-existence, replacement, admixture or differentiation [2], [4], [5]. For instance, recent detailed studies of secondary contact zones between morphologically cryptic lineages have revealed either significant pre- or post-mating reproductive isolation, shedding light on speciation processes and species boundaries [e.g. 6], [7]–[9].

A key mechanism influencing patterns of gene flow is divergence in pre-copulatory sexual selection (intra-sexual competition and mate choice), which may be generated by divergence of competitive abilities or mate preferences in allopatry or by reinforcement after contact [4]. Differences in mate preferences or competitive ability have been identified in several avian hybrid zones in which the lineages differ markedly in secondary sexual traits such as song structure or plumage [e.g., 10], [11]–[16]. These differences in pre-copulatory sexual selection have been central to explaining patterns of hybrid zone movement or stability and concordance between clines in genetic and morphological characters [10]–[14]. Divergence in aggressive or mating behaviour, however, may be associated with traits such as pheromones that may be obvious to the animals involved, but less obvious to researchers. For this reason, assessing variation in social and sexual behaviour can shed light on the potential evolutionary dynamics of cryptic hybrid zones.

Another important factor potentially influencing both the dynamics of hybrid zones and pre-copulatory sexual selection is interspecific interactions, particularly those between parasites and their hosts [17], [18]. Parasites may differentially impact host lineages due to lineage differences in immunocompetence and genetic diversity associated with parasite resistance, differences in physiology or behaviour and environmental differences affecting the prevalence of parasites or their intermediate hosts [19]. This in turn can affect competitive interactions or mate choice decisions [18]. Host-parasite interactions can potentially contribute to hybrid zone movement or lineage replacement if one lineage has a fitness advantage due to parasite resistance [17], [20], [21]. They may also contribute to either reinforcement or hybrid speciation when hybrids have inferior or superior parasite resistance respectively [17], [20], [22]. Although several studies have compared parasite susceptibility of hybrids to parental species [reviewed in 17], [20], [22], few have compared the levels of parasite infestation between genetically divergent intraspecific host lineages [but see 19].

**Figure 1:**
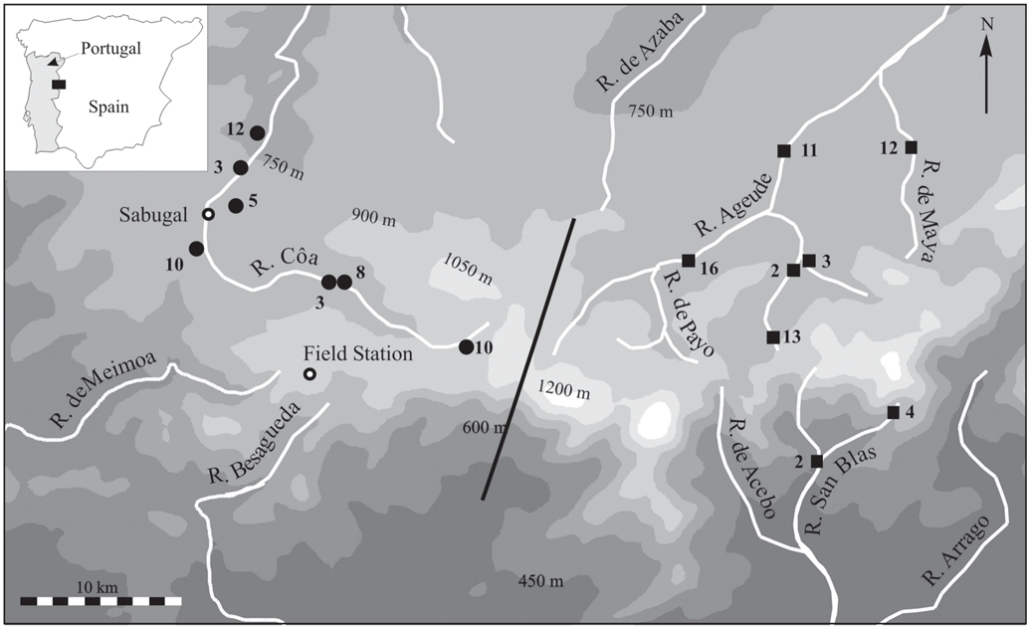
Elevation map of sampling localities with sample sizes for each locality. Filled circles and squares represent sites with MtDNA of the western and eastern lineages respectively, with the centre of the hybrid zone indicated by the black line. The inset shows the position of the study area (black rectangle) on the Iberian Peninsula. Sampling localities are located along rivers (white lines) as the species is almost exclusively riparian.

A hybrid zone exists between two genetically divergent yet morphologically cryptic lineages of the Iberian endemic lizard, *Lacerta schreiberi*. Genetic data indicate that the two lineages of *L. schreiberi* probably diverged as early as the Pliocene and persisted in separate glacial refugia, one in northwestern Iberia and the other in the Spanish Central System [9], [23]. At a broad spatial scale, nuclear data have revealed evidence for repeated historical admixture between the two lineages [9]. *Lacerta schreiberi's* current range corresponds to the Atlantic climatic envelope of the west coast of Portugal. However, a long corridor of this climate envelope penetrates into the Mediterranean climate of Spain through its Central System mountain range. This entire portion of Iberia is thought to have been temperate climate forest steppe at the last glacial maximum. At the centre of the current corridor of Atlantic climate phylogeographic studies have identified a sharp cline in mitochondrial DNA [23], and also clines in nuclear markers [9 both proteins and microsatellites], [24], [25]. The current hybrid zone between these genetic backgrounds is centred on a watershed dividing the western and eastern portions of the corridor [26] (Figure 1), correlated change at autosomal and mitochondrial markers being as expected for a female-heterozygous chromosomal sex determination hybrid zone [27]. The watershed potentially represents an environmental restriction on gene flow, as *L. schreiberi* shows a strong preference for riparian habitats [28]. Cline movement may nevertheless be expected if one genetic background has a net fitness advantage over the other [1], [29], potentially mediated by differences in male competitive ability or female mate preferences [e.g., 10], [16], [30]. Assessing the relative influence of environmental barriers, prezygotic barriers and fitness differentials across this hybrid zone would allow deeper insight into the evolutionary process at play when vicars meet [2], [4], [8].

Eastern and western habitats form approximate mirror images in altitude profile within the Atlantic climatic corridor, dropping gradually away from the central watershed. Here, we test whether males from the eastern and western *L. schreiberi* genetic backgrounds can be differentiated based on a combination of male traits that potentially influence patterns of mating success in lizards: body size, head dimensions and coloration [31], [32]. We also test whether the lineages differ in prevalence of ectoparasites (ticks) and endoparasites (haemogregarines). In lizards, pre-copulatory sexual selection occurs most often via the mechanism of male-male competition [32]–[34]. Male *L. schreiberi* compete over access to mates and guard mates after copulation so that competitive ability is likely to have a significant influence on male fitness [35]. Thus, we tested whether males of the two lineages differed in their aggressive behaviour or competitive ability and whether genetic background, phenotypic traits or parasite load were predictors of contest success and aggressive behaviour in *L. schreiberi*.

## Methods

We sampled populations of *Lacerta schreiberi* from the Central System mountains of the Iberian Peninsula. During the breeding season (April 20–May 15 2006), we caught 116 males (53 western and 63 eastern) on either side of the watershed (Figure 1), returned them to the field station (Malcata Nature Reserve; Figure 1), measured male traits and subsequently released them at their precise site of capture (marked with GPS and flagging tape). Individuals were sampled at eight sites for each lineage (see Figure 1 for sample sites and sizes), which covered elevations from approximately 600–950 m a.s.l. (western sites mean elevation = 760±60 m S.D.; eastern sites mean elevation = 790±55 m S.D.; ANOVA: F_1_ = 5.87, P = 0.02). At the field station, males were housed temporarily in enclosures approx. 40×25×30 cm (length×width×height) containing bark substrate, a leafy branch and concave ceramic tile for shelter and water dish. Lizards were kept for no longer than 5 days in captivity.

### Genetic backgrounds

We sampled individuals from localities between 25 and 15 km west, and between 10 and 25 km east of the previously identified mtDNA transition [9], [24], [25]. We verified that our sampling localities fell on either side of the transition by assaying mitochondrial haplotypes. We took a DNA sample (tail tip) from each individual and sequenced 850 base pairs of the mitochondrial cytochrome b gene. Protocols follow those in [9], [24], [25]. In western and eastern localities, the sampled frequency of introgressed mtDNA was 0/53, and 0/63 respectively, confirming our choice of sample localities as bracketing the mtDNA cline.

### Morphology and parasite load

We measured several morphological traits, chosen for their importance in determining contest success in other lizards [31], [32]: body size (snout-vent length, SVL, to the nearest 0.1 mm), head depth (deepest point of head, in line with tympanum), jaw length (angle of the jaw to the tip of the snout) and body mass. We measured mass to the nearest gram and head dimensions to the nearest 0.01 mm. We converted mass, head depth and jaw length to size-free variables by taking the residuals of these variables regressed against SVL.

Lizards carried two types of ectoparasites: ticks (*Ixodes ricinus*) and mites (*Ophionyssus schreibericolus*) [36]. We counted the number of ticks on each individual and collected a sub-sample to check their developmental stages. The majority were nymphs (73%) and the remaining ticks were at the larval stage (27%). As mites dislodged easily making counts unreliable, we focused on the total number of ticks (irrespective of developmental stage) per individual as a measure of relative ectoparasite load (mean = 12; range = 0–81). We scored the presence or absence of intraerythrocytic haemogregarines from blood smears. Haemogregarines are apicomplexan protozoans with heteroxenous life cycles, alternating between vertebrate and invertebrate hosts [37]. In the vertebrate intermediate host there is an obligate phase of intra-erythrocytic development of the parasite. All haemogregarines undergo the majority of their development within a definitive invertebrate host, which also serves as a vector. As it is difficult to classify haemogregarines to species level based solely on the gametocytes in vertebrate erythrocytes [38], we classified these parasites to family level for the purposes of this study. Using a drop of blood collected during tail-tipping, we made blood smears on microscope slides. The blood smears were air dried, fixed in absolute methanol for five minutes, then stained with Giemsa (diluted 1∶10 in buffered water, pH 7) for 20 minutes and examined with an Olympus BX 41 microscope at ×400 magnification [39]. We obtained adequate blood smears for 101 of the 116 males (50 western and 51 eastern). Presence of haemogregarines was affirmed by checking of at least 10,000 erythrocytes per smear [39].

### Colour and colorimetrics

**Figure 2:**
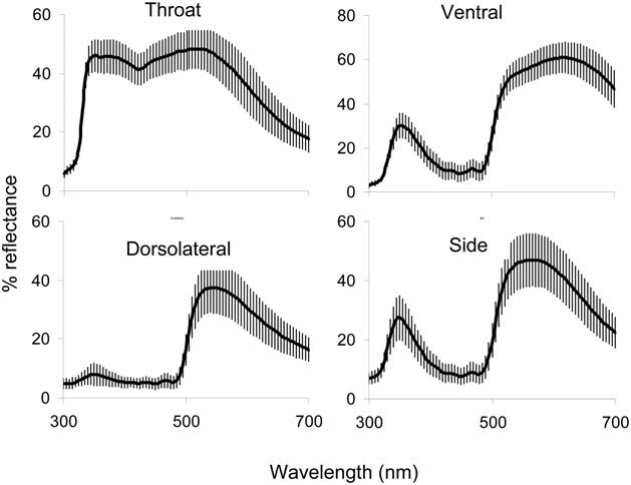
Mean reflectance spectra for each male body region (N = 116). Bars are the standard deviation around the mean. Note the substantial UV reflectance (300–400 nm) of the throat, side and ventral region.

We measured the reflectance of six body regions using an Ocean Optics SD2000 spectroradiometer and PX2 light source (Ocean Optics, Florida, USA) with illumination at 45° relative to the surface and reflectance measured at the same angle. Readings were taken from a 3 mm diameter area at a constant distance from the surface in a darkened room. Measurements were relative to a 99% WS-1 white reflectance standard. Measurements of lizards were taken from four body regions (Figure 2): green dorsolateral region, yellow-green side, pale blue throat (mean of proximal and distal throat measurements) and yellow venter (mean of proximal and distal measurements). Because we were simply interested in male colour variation and do not have information on lacertid visual systems, we calculated three commonly used metrics to summarize variation in spectral reflectance curves. We calculated spectral location as the wavelength of maximum reflectance, spectral intensity as the mean reflectance across all wavelengths (320–700 nm) and spectral purity using the formula [R_320−λ(R50)_−R_λ(R50)−700_]/R_320–700_] where λ_(R50)_ is the wavelength half way between the minimum and maximum reflectance [40], [41]. These measures are analogous to the human perceptions of hue, brightness and chroma respectively and we refer to them as such for simplicity.

### Behavioural trials

We conducted male-male contests using a subset of these males (N = 68, 34 of each genetic background), with each male used in one contest only. The average size of males used in contests was 92.3 mm±9.0 mm S.D. We size-matched males from the two genetic backgrounds as closely as possible to enable us to detect subtle differences in competitive ability based on genetic lineage or morphological traits. Males were size-matched to within an average of 3.9±5.3% S.D. of SVL. All experiments were conducted in accordance with the Association for the Study of Animal Behaviour ‘Guidelines for the Treatment of Animals in Behavioural Research and Teaching’ and animal ethics guidelines of the Portuguese Institute of Nature Conservation.

Contests were conducted in a neutral arena 80×80×40 cm (length×width×height), outdoors under sunny conditions when lizards were naturally active. Contests were video-taped and lasted approximately 30 min. From the videos, we counted the number of approaches, number of chases, number of bites, duration of bites, number of arm waves (slow circumduction of left or right front limb) and number of times each lizard fled. Approaches, chases and bites were aggressive behaviours while arm-waving and fleeing were submissive behaviours. We also calculated a weighted index of aggression based on aggressive behaviours only as (aggression = 1×approaches+2×chases+3×bites) reflecting increasing energy expenditure and risk of harm [42]–[45].

### Statistical analysis

We tested whether males of the two genetic backgrounds could be differentiated based on morphometric measurements (including body size), coloration and ectoparasite load using a discriminant function analysis with genetic background (eastern or western) as the classification variable (N = 115 due to missing data for one individual). We first applied a stepwise discriminant procedure (PROC STEPDISC, SAS 9.1) to identify the combination of variables that best differentiates the classes. Next, we used these variables in a discriminant function analysis (PROC DISCRIM SAS 9.1), which derives a function (criterion) to classify individuals into each group. To account for variation among sites, we tested whether each of the variables used in the discriminant function analysis differed significantly between the genetic backgrounds using a general linear mixed model (GLMM, PROC MIXED SAS 9.1) with site nested within genetic background as a random factor. We also tested whether any of the variables were correlated with altitude (Pearson correlation, PROC CORR SAS 9.1).

Presence of blood parasites was not included in the discriminant function analysis as it is a categorical variable. We tested whether the lineages differed in prevalence of haemogregarines using a GLMM (PROC MIXED SAS 9.1) with site nested within genetic background as a random factor.

We tested whether genetic background or male traits predicted contest success and aggressive behaviour. For this analysis, we used a multivariate GLMM (PROC MIXED, SAS 9.1) with a random factor identifying trials to account for non-independence of males within the same trial. We derived five separate models, one for each of the following dependent variables: 1) contest outcome (won/lost) 2) aggression index; 3) number of bites; 4) duration of bites and 5) number of arm waves. We chose the latter three variables because biting represented the most common aggressive behaviour while arm waving was the most common submissive behaviour. For each model, the independent variables (predictors) were genetic background, SVL, body condition (the residuals of mass regressed against SVL), relative head depth, relative jaw length, parasite load (number of ticks and presence or absence of haemogregarines) and coloration (spectral location, spectral purity and spectral intensity of the dorsolateral region, side, average yellow ventral area and average blue throat colour). Behavioural variables and number of ticks were log-transformed [log (x+1)] to meet model assumptions. For each model, we applied stepwise model selection to identify the subset of traits that best predicted each behavioural variable.

Finally, we examined the inter-correlation among male traits using Pearson correlations (PROC CORR SAS 9.1).

## Results

### Lineage differentiation

**Figure 3:**
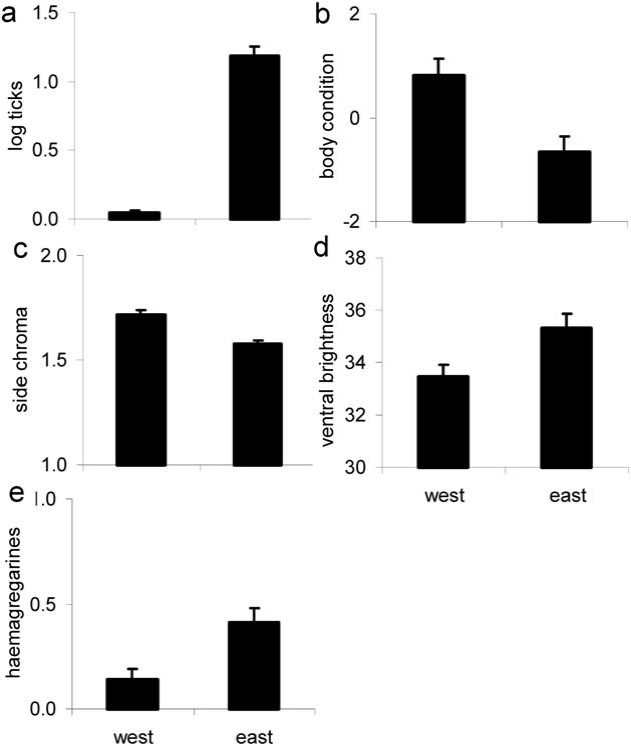
Differences between males from the two genetic backgrounds (N = 116). a)–d) variables identified in the multivariate stepwise discriminant analysis that were also significantly different in univariate tests a) number of ticks; b) body condition; c) chroma of the UV-green side and d) brightness of the yellow belly. Hue of the side (not shown) was significant in the multivariate model but not in univariate tests (see text). e) frequency of haemogregarines (N = 101).

**Figure 4:**
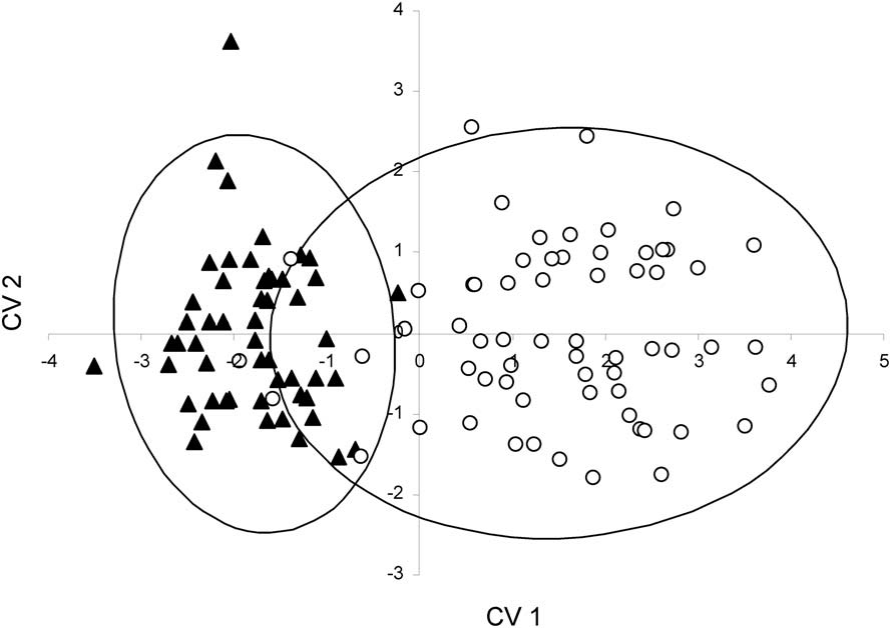
Plot of canonical variates 1 and 2 from the discriminant function analysis showing the position of each male from the two genetic lineages (N = 116). Filled triangles represent the western lineage and unfilled circles represent the eastern lineage. Ellipses represent 95% confidence ellipses. CV1 differentiates individuals based primarily on tick load, spectral purity of the side and body condition (standardised canonical correlation between CV1 and tick load = 0.95; side spectral purity = −0.46; body condition = −0.36). CV 2 discriminates between populations primarily on ventral brightness (standardised correlation = 0.95).

Males belonging to the two genetic backgrounds can be differentiated based on morphometric and colour traits as well as parasite load. The stepwise discriminant analysis identified five traits (from 16 original variables: SVL, body condition, relative head depth, relative jaw length, number of ticks, presence of haemogregarines, and coloration (spectral location, purity and intensity of four body regions)) that differentiated males based on genetic background. The five traits were tick load (F_1,110_ = 169.32, P<0.0001), body condition (F_1,110_ = 3.33, P = 0.07), chroma of the UV-green side (F_1,110_ = 15.43, P = 0.0001), hue of the side (F_1,110_ = 6.98, P = 0.009) and brightness of the yellow belly, (F_1,110_ = 4.5, P = 0.036). Compared to eastern males, western males had significantly fewer ticks, were in better body condition, had greener sides (hue shifted to longer wavelengths), higher chroma of the side and darker yellow belly (Figure 3). Multivariate discrimination based on the five traits was highly significant (Wilks' λ = 0.27, F_5,110_ = 59.86, P = <0.0001) and classification success was high (western background 100% and eastern background 90.48%) with moderate overlap between backgrounds (Figure 4).

All these traits but the hue of the side differed significantly between the lineages in univariate tests accounting for variation among sites (Figure 3; GLMMs: number of ticks: F_1,15_ = 32.1, P<0.0001; body condition: F_1,15_ = 4.52, P = 0.05; side chroma: F_1,15_ = 15.87, P 0.0012; side hue: F_1,15_ = 1.21, P = 0.29; ventral brightness: F_1,15_ = 5.8, P = 0.03). Tick load increased with increasing altitude (R = 0.42, P<0.0001) and body condition decreased with increasing altitude (R = −0.24, P = 0.01). The remaining three traits were uncorrelated with altitude (side chroma: R = −0.07, P = 0.5; side hue: R = −0.1, P = 0.33; ventral brightness: R = 0.16, P = 0.11). Genetic background remained a significant predictor of number of ticks (F_1,13_ = 43.25, P<0.0001) and body condition (F_1,13_ = 4.52, P = 0.05) when altitude was included in models as a covariate. The genetic backgrounds also differed in the occurrence of haemogregarines (F_1,16_ = 5.8, P = 0.02) with the western background having a significantly lower frequency (Figure 3e). Similar to the number of ticks, prevalence of haemogregarines was significantly higher at higher elevations (F_1,87_ = 7.73, P = 0.007) but genetic background remained a significant predictor of haemogregarine prevalence when altitude was included in models as a covariate (F_1,14_ = 9.47, P = 0.008).

### Predictors of male contest success and aggressive behaviour

Genetic background did not predict contest outcome or any aspect of aggressive behaviour. The only predictor of contest outcome was relative head depth: winners had deeper heads relative to their body size (F_1,10_ = 6.36, P = 0.03). Only three variables were retained in the final model predicting aggression index: body size (F_df_ = 15.35_1,8_ P = 0.004), ventral chroma (F_1,8_ = 4.64, P = 0.06) and dorsolateral chroma (F_1,9_ = 18.63, P = 0.003). More aggressive males were larger, had lower dorsolateral chroma but slightly higher ventral chroma, that is, they showed a less saturated green dorsolateral region but a slightly more saturated yellow belly. Predictors of contest outcome and aggression remained qualitatively unchanged with or without inclusion of genetic background in the models.

Bite number was positively associated with body size (F_1,9_ = 24.17, P<0.001) and negatively associated with dorsolateral chroma (F_1,9_ = 11.83, P = 0.007), as was bite duration (SVL: F_1,9_ = 16.33, P<0.001; dorsolateral chroma: F_1,9_ = 8.93, P = 0.015). The most common submissive behaviour apart from fleeing from the aggressor was arm waving. Frequency of this behaviour was negatively associated with relative head depth (F_1,9_ = 6.03, P = 0.037) and ventral chroma (F_1,9_ = 5.57, P = 0.043).

### Correlations among traits

There were some correlations among colour variables, in particular the colours of the dorsolateral and side regions (Table 1). All correlations, however, were moderate to weak (r≤0.5, Table 1). Tick load was weakly negatively correlated with body condition and spectral purity of the side (r = 0.27 and 0.26 respectively, Table 1) but no other morphological or colour traits. The prevalence of haemogregarines was not associated with tick load, morphology or coloration after sequential Bonferroni correction for multiple tests. Thus, more parasitized males were in poorer body condition but were no less intensely coloured. Moreover, body condition was uncorrelated with coloration, although males in better condition had slightly larger heads (deeper heads and longer jaws).

**Table 1:** Relationships among male traits (N = 116 for all traits except haemogregarines for which N = 101).

	Haema-gregarines	ticks	SVL	Mass	head depth	jaw	dorso-lat SL	side SL	throat SL	vent SL	dorso-lat SP	side SP	throat SP	vent SP	dorso-lat SI	side SI	throat SI	vent SI
Haemogregarines	-	0.03	0.42	0.09	0.64	0.67	0.01	0.03	0.18	0.06	0.46	0.19	0.67	0.94	0.11	0.78	0.72	0.67
ticks	4.71	-	0.35	**0.004**	0.15	0.12	0.19	0.35	0.34	0.48	0.03	**0.003**	0.10	0.29	0.87	0.24	0.73	0.22
SVL	0.66	0.09	-	0.37	**0.002**	**<0.0001**	**<0.001**	**<0.0001**	0.24	0.10	0.61	0.05	0.18	**<0.001**	0.46	0.12	**0.003**	**<0.0001**
Mass	3.01	**−0.26**	0.08	-	**<0.001**	**0.004**	0.91	0.74	0.08	0.63	0.73	0.83	0.89	0.15	0.56	0.34	0.07	0.05
Head depth	0.22	**−**0.13	**0.28**	**0.31**	-	**<0.001**	0.94	0.98	0.36	0.88	0.91	0.69	0.49	0.22	0.79	0.28	0.79	0.12
Jaw length	0.18	**−**0.15	**0.39**	**0.26**	**0.34**	-	**0.006**	0.04	0.95	0.42	0.97	0.62	0.14	**0.001**	0.93	0.47	0.12	0.07
dorso-lat SL	6.22	**−**0.04	**−0.35**	0.01	−0.01	**−0.26**	-	**<0.0001**	0.32	0.01	**<0.0001**	0.76	0.06	0.11	0.20	0.64	0.38	0.36
side SL	4.70	−0.09	**−0.47**	0.03	−0.002	−0.19	**0.52**	-	0.10	**<0.0001**	0.008	0.007	0.14	0.05	0.09	0.24	0.02	0.05
throat SL	1.86	−0.09	−0.11	−0.16	0.09	0.01	0.09	0.15	-	0.004	0.55	0.52	0.69	0.22	**0.001**	0.05	**<0.0001**	0.01
vent SL	3.56	0.07	−0.15	−0.04	0.01	−0.08	0.23	**0.50**	0.27	-	0.57	0.08	**<0.0001**	0.89	0.02	0.34	**0.0001**	0.01
dorso-lat SP	0.55	−0.21	0.05	0.03	−0.01	−0.003	**−0.43**	−0.24	0.06	0.05	-	**0.0008**	0.29	0.02	0.55	0.75	0.26	0.37
side SP	1.75	**−0.27**	0.18	0.02	0.04	0.05	−0.03	−0.25	0.06	0.16	**0.31**	-	**0.002**	0.05	0.09	0.03	0.65	0.01
throat SP	0.19	−0.15	0.12	−0.01	0.06	0.14	−0.17	−0.14	0.04	**−0.38**	0.10	**0.28**	-	**0.0007**	0.67	0.40	0.08	0.01
vent SP	0.01	−0.10	**0.31**	0.13	0.12	**0.30**	−0.15	−0.18	−0.11	−0.01	0.22	0.18	**0.31**	-	0.86	0.09	0.10	**0.0002**
dorso-lat SI	2.65	−0.01	−0.07	0.06	0.02	0.01	0.12	0.16	**0.30**	0.22	−0.06	0.16	0.04	0.02	-	**0.0005**	**0.002**	0.06
side SI	0.08	0.11	−0.14	−0.09	0.10	−0.07	−0.04	0.11	0.18	0.09	0.03	−0.20	−0.08	−0.16	**0.32**	-	**0.004**	**0.005**
throat SI	0.12	−0.03	**−0.27**	−0.17	−0.02	−0.14	0.08	0.22	**0.50**	**0.35**	0.10	0.04	−0.16	−0.15	**0.29**	**0.27**	-	**<0.0001**
vent SI	0.18	0.12	**−0.4**	−0.18	−0.14	−0.17	0.09	0.18	0.23	0.23	−0.08	−0.23	−0.25	**−0.34**	0.17	**0.26**	**0.46**	-

Pearson correlation coefficients are below the diagonal and P values are above the diagonal. As haemogregarines is a categorical variable, values below the diagonal are F-statistics from a general linear model. Mass, head depth and jaw length are size-corrected residuals. SL stands for spectral location, SP for spectral purity and SI for spectral intensity. Relationships that are significant after sequential Bonferroni correction for multiple tests are in bold.

**Figure 5:**
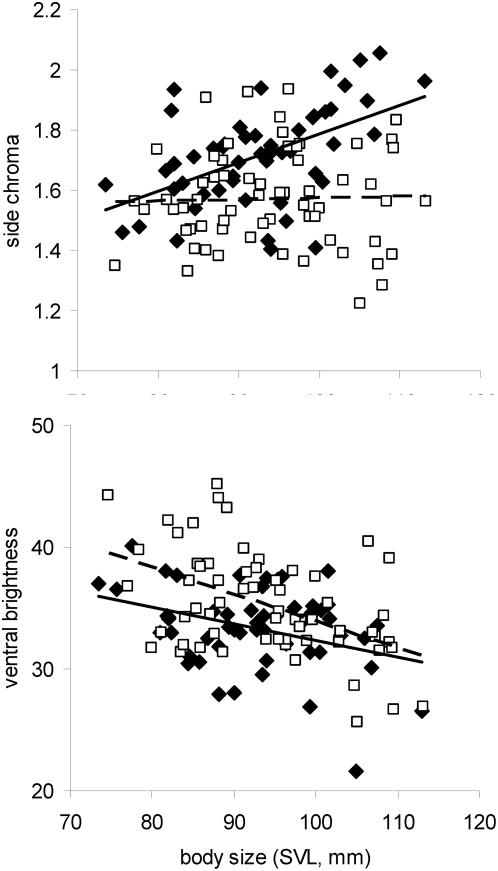
Relationship between body size (SVL) and a) side chroma; b) ventral brightness. Filled diamonds and solid regression line represent the western genetic background while empty squares and dotted regression line represent the eastern background. There is a significant difference between the genetic backgrounds in the slopes in a) and in the intercept in b); see text. Note that there is no overall difference in body size between eastern and western males (F_1_ = 0.89, P = 0.35).

Body size was correlated with relative head depth, relative jaw length, hue of the dorsolateral and side regions, ventral chroma and throat and ventral brightness (Table 1). Larger males had relatively deeper heads and longer jaws for their size, yellower-green sides and dorsolateral body regions, a more saturated yellow venter, darker blue throats and darker yellow bellies. Thus, body size (SVL), which strongly predicts aggression, is positively associated with traits that predict contest outcome (e.g. head depth) as well as traits that differentiate the genetic backgrounds (e.g. ventral brightness). To further explore the relationship between body size and the three colour traits differentiating the genetic backgrounds (side chroma, side hue and ventral brightness), we performed ANCOVAs (PROC GLM) to test for differences between the backgrounds in the slope or intercept of these relationships. There was a significant difference in the slope for side chroma (F_1_ = 7.98, P = 0.006; Figure 5a). Body size was strongly associated with side chroma in western males (F_1_ = 18.15, P<0.0001) but not the more parasitized eastern males (F_1_ = 0.05, P = 0.82). There was no significant difference in slope for the relationship between body size and either side hue (F_1_ = 0.9, P = 0.35) or ventral brightness (F_1_ = 1.29, P = 0.23) and there was a significant difference in intercept only for the latter (F_1_ = 10.78, P = 0.001; Figure 5b).

## Discussion

Our results show that although the two genetic lineages forming the *L. schreiberi* hybrid zone appear largely morphologically cryptic, once identified by their genetics they can be differentiated based on a combination of phenotypic traits, namely body condition, chroma and hue of the UV-green side and brightness of the yellow belly. They also differ in parasite load (ticks and haemogregarines), with the western lineage being markedly less parasitized. Despite the differences, males of the two genetic backgrounds did not differ in aggressive behaviour or competitive ability, which were also un-related to parasite load. Instead, contest outcome (win/lose) was predicted by head size while aggression was positively associated with body size and dorso-lateral and ventral chroma. Taken together, these results suggest that although there are potential fitness differentials across the hybrid zone associated with both morphological differences and host-parasite interactions, these are unlikely to be directly mediated by differences in competitive ability or aggressive behaviour.

### Differentiation in morphology and parasite load

The phenotypic differences we observed between males of the two genetic backgrounds have two potential explanations. This first is that they reflect heritable divergence (that is, the traits have a genetic basis or may be epigenetically inherited), and the second is that they reflect a phenotypically plastic response to environmental differences on either side of the hybrid zone. Similarly, the differences in parasite load could be explained by heritable differences in immunocompetence or environmentally based differences in the abundance of parasites or their intermediate hosts. Our results suggest that environmental differences are likely to at least partially explain differences in parasite load and body condition, but are unlikely to explain colour differences, which are instead likely to reflect heritable divergence.

At a broad scale, habitats on the eastern side of the range tend to be more humid and more densely vegetated compared to the western side of the range although there are no obvious differences in the riparian micro-habitats preferred by this species [26]. Moreover there was a small, but significant difference in the elevation of sites sampled for the eastern and western populations (with western sites being at slightly lower mean elevations) and elevation was significantly positively associated with the prevalence of both ticks and haemogregarines. The possibility therefore exists that the habitats of the two populations differ in the abundance of parasites or the abundance or community composition of their hosts. *Ixodes ricinus* has catholic feeding habits and can be found on a wide range of vertebrate hosts [46] while the best known haemogregarines of European lacertid lizards (genus *Karyolysus*) rely on mites as definitive hosts [38], [47], [48]. Variation in farming practices or ecotype across the study area (also potentially correlated with elevation) may lead to higher densities of ticks or mites in the east and thus explain the difference in parasite loads in the lizards. However, the differences in parasite load could also reflect genetically-based differences in immunocompetence or host behaviour, given that prevalence of two types of parasite with different host specificity and life cycle shows the same pattern across the hybrid zone. The two types of parasite are not directly linked: *Ixodes ricinus* is not known to be a vector of any haemogregarine parasite and is well studied in nature because it is a vector of Lyme disease, menigoencephalitis and other diseases affecting humans. Experiments assessing susceptibility of the two genetic backgrounds to parasites and testing for behavioural differences that may influence parasite infestation are needed to assess the relative roles of environment and heritable divergence.

In many animals, parasite load influences body condition and colour expression [e.g. 49], [50], [51] but see [52]–[54]. Therefore, the differences in body condition and coloration between the two genetic backgrounds could conceivably be a consequence of habitat-associated differences in parasite load. However, in *L. schreiberi*, the prevalence of blood parasites was not associated with body condition or coloration, while ectoparasite load was weakly correlated with body condition and the spectral purity of the side but not the hue of the side and intensity of ventral coloration (or any other colour trait). Furthermore, colour traits were uncorrelated with elevation. Thus, environmentally mediated differences in parasite load are unlikely to explain differences in coloration although they could explain the differences in body condition. Instead, we suggest that coloration differences are more likely to reflect heritable divergence because coloration is a key adaptive trait influencing predation risk [55], [56] and potentially mating success [57], [58].

### Contest success and aggressive behaviour

Given that western males are in better condition, have fewer parasites and are more intensely coloured, there is potentially a male fitness differential across the hybrid zone if these traits influence survival or reproductive success. We found no evidence that males from the two genetic backgrounds differed in their competitive ability or that the colour traits differentiating them (side hue and chroma and ventral brightness) predicted aggressive behaviour. Instead, the most aggressive males were larger, had slightly yellower bellies (higher ventral chroma) and more very fine black dorso-lateral speckling, resulting in lower spectral purity of this body region. Males may therefore use coloration to assess the size and potential competitive ability of opponents. This is likely given that the majority of encounters do not escalate to physical contact and contests are settled by assessment of rivals from a distance in structurally complex riparian habitat [28], [35, Godinho and Brito, pers. obs.]

Field data indicate that in *L. schreiberi*, larger males win more contests when mate-guarding and have greater mating frequency, number of mates and mating success [35]. In our sample of males, body size was correlated with several aspects of coloration including the three colour traits that differentiate the genetic backgrounds. Even though males from the two genetic backgrounds did not differ in aggressive behaviour or competitive ability, the colour differences between them may have fitness consequences via their relationship with body size if colour traits are used by males to assess the size of potential rivals and/or by females to assess the size of potential mates. Although there was no overall difference in body size between eastern and western males, the slope and intercept of the relationship between body size and side chroma and ventral brightness respectively differed between the genetic backgrounds. Consequently, based on coloration, an eastern lizard might judge a western male to be larger than he actually is, potentially resulting in a fitness differential across the hybrid zone.

Although coloration predicted variation in male aggressive behaviour, it did not predict contest outcome. Instead, contest outcome was predicted by head size, as in numerous other lizards [e.g. 59], [60], [61], including lacertids [62]. In lizards, head size is associated with bite force, which influences the outcome of escalated contests [63], [64]. We suggest that *L. schreiberi* may use a combination of size, coloration, and behavioural displays [and potentially olfactory cues, see 65], [66] to assess the competitive ability of opponents. However, the outcome of escalated contests, which are likely to be more frequent in staged encounters than in nature, may be largely determined by head size and associated bite force.

### Conclusions

Overall, we found no evidence for a clear competitive advantage of one lineage over the other, unlike in several avian hybrid zones [e.g. white-collared and golden-collared manakins [but see 13], [15], hermit and Townsend's warblers [14], [30] and black-capped and Carolina chickadees [10]]. Results of these staged contests between size matched individuals, however, should ideally be confirmed with field data reflecting the full range of variation in fitness between males. Furthermore, we cannot exclude the possibility of habitat-genotype interactions, which may be apparent in the contact zone but not in staged contests. Nevertheless, we found that males from the two genetic backgrounds differ in subtle aspects of coloration that are correlated with body size, which influences aggressive behaviour and competitive ability. If, as a result, there is a fitness differential favouring the more intensely coloured western males, we would predict net male-mediated gene flow from west to east across the contact zone. The nuclear cline would shift east, leaving the matrilineally inherited mitochondrial cline behind. This scenario is consistent with findings for mitochondrial and nuclear loci at the current contact [26, Baird and Godinho unpublished data]. By contrast, if western males have a fitness advantage only due to environmental change across the hybrid zone resulting in lower parasite load and better body condition, we would predict the nuclear and mitochondrial clines to remain coincident. The fitness differential would instead cause stable, asymmetric clines. A more detailed characterization of nuclear genetic and phenotypic variation across the hybrid zone is required to distinguish these scenarios.
